# Proof of biased behavior of Normalized Mutual Information

**DOI:** 10.1038/s41598-024-59073-9

**Published:** 2024-04-19

**Authors:** Amin Mahmoudi, Dariusz Jemielniak

**Affiliations:** Management in Networked and Digital Societies (MINDS) Department, Kozminski University, Warsaw, Poland

**Keywords:** Computational science, Computer science

## Abstract

The Normalized Mutual Information (NMI) metric is widely utilized in the evaluation of clustering and community detection algorithms. This study explores the performance of NMI, specifically examining its performance in relation to the quantity of communities, and uncovers a significant drawback associated with the metric's behavior as the number of communities increases. Our findings reveal a pronounced bias in the NMI as the number of communities escalates. While previous studies have noted this biased behavior, they have not provided a formal proof and have not addressed the causation of this problem, leaving a gap in the existing literature. In this study, we fill this gap by employing a mathematical approach to formally demonstrate why NMI exhibits biased behavior, thereby establishing its unsuitability as a metric for evaluating clustering and community detection algorithms. Crucially, our study exposes the vulnerability of entropy-based metrics that employ logarithmic functions to similar bias.

## Introduction

### Background

Community detection (CD) within social networks has emerged as a pivotal area of research, given its potential to unravel intricate patterns of interaction and group dynamics. It is used in many disciplines, including biology^1^, criminology^2^, economics^3^, and urban planning^4^, to mention just a few examples. In particular, this topic has also emerged as a critical field in the battle against disinformation. Social networks are often the primary conduits for the spread of disinformation, with communities within these networks playing a significant role in the dissemination and amplification of misleading content. By identifying and understanding these communities, we can gain valuable insights into the dynamics of disinformation spread, enabling more effective interventions^5,6^. It is essential, as the rapid spread of medical^7,8^, political^9,10^, social^11–13^, as well as scientific^14–16^ misinformation and disinformation are among the greatest civilization challenges of our times^17–19^.

Research has shown that disinformation tends to spread rapidly within tight-knit communities and is often characterized by homogenous beliefs and high levels of trust among members^20^. These communities can act as echo chambers, reinforcing and amplifying disinformation and using more and more sophisticated strategies for masking their agendas^21^. By employing CD algorithms, we can identify these communities and understand their structure and behavior, providing a basis for targeted, community-specific strategies to combat disinformation. As such, CD in social networks is not only a theoretical exercise but also a practical tool in the fight against disinformation. However, even though many new emerging approaches have been tested^22–25^, we are still far from an optimal approach.

A plethora of algorithms have been developed to enhance the accuracy of CD, yet comprehensive and diverse sets of metrics for evaluating these algorithms are lacking. Predominantly, metrics such as modularity, conductance, pairwise F-measure (PWF), NMI, variation of information (VI), purity, and adjusted rand index (ARI) have been employed to assess the performance of CD algorithms. These metrics, which were originally designed for evaluating clustering techniques, have been adapted for CD due to the conceptual similarities between clustering and community detection^26^.

Certain metrics, such as modularity, operate based on the internal structure of communities identified by a specific algorithm, independent of the availability of ground truth^27^. Conversely, metrics such as the NMI, ARI, VI, purity, and F-measure necessitate the availability of ground truth for deployment^28^. Regardless of the accessibility of ground truth, each metric is subject to a resolution limit^29^, which is a factor that has been highlighted and well established in existing research^30–34^.

### Motivation

Predominantly, external metrics (those reliant on ground truth) construct a contingency matrix (table), where each cell represents the intersection of members between actual classes and detected communities. In some instances, the accuracy of CD algorithms is evaluated solely based on the number of true positive members. However, an effective CD algorithm should take into account two primary aspects of communities: distribution and joint membership. Therefore, the evaluation metric for CD algorithms must be capable of discerning the distribution of communities and joint members in relation to the ground truth. This implies that the critical factor is not merely identifying joint members but also accurately determining the number of communities relative to the ground truth^35^.

Despite the identification of biased behavior in these measures by several researchers^32–34^, a mathematical explanation for this issue has not been adequately addressed. Most reports suggest that this bias is due to the finite size effect. In this study, we aim to formally demonstrate why the NMI metric exhibits bias. To achieve this, we first present the results of NMI across 40 scenarios (representing different community numbers assumed to be detected by a specific algorithm) and compare them with five other well-established measures. Subsequently, we dissected the NMI formula and ultimately proved that this formula inherently leads to biased behavior^36^.

### Contribution

Community detection is a critical concern spanning diverse scientific disciplines, including biology, health, social networks, politics, targeted marketing, recommender systems, link prediction, and criminology^37^. As such, the accuracy of community detection algorithms is of paramount importance, and the evaluation metric for these algorithms assumes even greater significance. Given the widespread use of the NMI in evaluating community detection algorithms, illuminating its biased behavior contributes significantly to fields that employ community detection studies. Moreover, substantiating this bias establishes the foundation for analyzing evaluation metrics of community detection algorithms that incorporate a logarithmic function^38^. It also opens the door to crafting new metrics while considering issues rooted in logarithmic functions.

The remainder of this paper is structured as follows: The subsequent section reviews the relevant literature in this domain. Then “Preliminaries and notations” are introduced. “Problem statement” delves into a detailed description of the NMI drawback. The proof of the NMI problem is presented in “Proof of biased behavior of NMI”. In “Case study”, we present a case study based on a real-world dataset and “Conclusion” concludes the paper with key findings and implications.

## Related works

CD algorithms aim to identify groups of nodes characterized by dense interconnections compared to the rest of the network^39,40^. Girvan and Newman^39^ introduced the modularity metric to evaluate the accuracy of communities detected by their algorithm, sparking the development of numerous algorithms based on this metric. However, Fortunato^26^ highlighted a resolution limit in the modularity metric, indicating its inability to detect small-sized communities. Cai et al.^31^ further demonstrated that maximizing modularity is an NP-hard problem and that a random network without any communities can achieve a high Q value. Chen, Nguyen, and Szymanski^41^ underscored the inconsistencies of the modularity metric, noting its tendency to favor either small or large communities in different scenarios. They proposed a new measure, modularity density, which combines modularity with split penalty and community density to circumvent the dual problems inherent in modularity.

The NMI was first considered a precise metric by Danon et al.^36^, who reported its sensitivity to errors in the community detection procedure. They consider ${Z}_{out}$ as the average number of links a node has to members of any other community, by increasing ${Z}_{out}$ NMI tends to be zero. Subsequent research has addressed the limitations of the NMI measure, with Romano et al.^42^ emphasizing the role of the number of clusters in the evaluation metrics. Amelio and Pizzuti^30^ argued that the NMI is not fair, as solutions with a high number of clusters receive disproportionately high NMI. Zhang^34^ demonstrated that the NMI is significantly affected by systematic errors due to finite network sizes and proposed the relative normalized mutual information (rNMI). Lai and Nardini^32^ introduced the corrected normalized mutual information (cNMI) to address the reverse finite size problem of the rNMI. Liu, Cheng, and Zhang^33^ highlighted the drawbacks of NMI and its improved versions, such as rNMI and cNMI, noting that these measures often overlook the importance of small communities. Rossetti, Pappalardo, and Rinzivillo^43^ introduced community precision and community recall to evaluate CD algorithms, addressing the high computational complexity of the NMI. Arab and Hasheminezhad^44^ also reported scalability problems with the NMI in large-scale data.

Other researchers have proposed alternative measures for evaluating community detection and clustering algorithms. Meilă^45^ introduced the variation of information (VI) metric, an entropy-based measure that operates based on mutual information. Wagner and Wagner^46^ categorized measures based on counting pairs, set overlaps, and mutual information and concluded that information theoretical measures outperform counting pairs and set overlaps measures. Santos and Embrechts^47^ utilized the ARI for cluster validation and feature selection. Yang and Leskovec^48^compared 13 measures for evaluating community detection algorithms, categorizing them into four groups and concluding that conductance and triad-participation-ratio have the best performance in identifying communities. Saltz, Prat-Pérez, and Dominguez-Sal^49^ introduced a new metric for the CD problem, weighted community clustering (WCC), which operates based on the distribution of triangles in the graph.

## Preliminaries and notations

### Normalized Mutual Information (NMI)

The NMI serves as a metric for assessing the performance of community detection algorithms. The NMI facilitates comparisons between two clusters or communities, yielding a value that ranges from 0 to 1. A higher value indicates a greater degree of similarity between two partitions or communities. As an external metric, the NMI necessitates the availability of class labels for computations, implying that the ground truth is required when employing this metric. The calculation of NMI is executed according to Eq. (1).

1 $$NMI\left(A,B\right)=\frac{2*I(A,B)}{[H\left(A\right)+H(B)]}$$

where $I(A,B)$ is mutual information and $H$ is the entropy as shown in Eqs. (2) and (3).

2 $$I\left(A,B\right)=\sum_{i=1}^{S}\sum_{j=1}^{R}p({community}_{j}\cap {class}_{i})log\frac{p({community}_{j}\cap {class}_{i})}{p({community}_{j}){p(class}_{i})}$$

$$=\sum_{i=1}^{S}\sum_{j=1}^{R}\frac{{|community}_{j}\cap {class}_{i}|}{N}log\frac{{|community}_{j}\cap {class}_{i}|N}{\left|{community}_{j}\right||{class}_{i}|}$$

3 $$H\left(A\right)=-\sum_{j=1}^{R}p\left({community}_{j}\right){\text{log}}p \left({community}_{j}\right)=-\sum_{j=1}^{R}\frac{{|community}_{j}|}{N}log\frac{{|community}_{j}|}{N}$$

The expansion of Eq. (1) with respect to (2) and (3) is Eq. (4)

4 $$NMI\left(A,B\right)=\frac{-2\sum_{i=1}^{S}\sum_{j=1}^{R}{C}_{ij}{\text{log}}\frac{{C}_{ij}N}{{C}_{i.}{C}_{.j}}}{\sum_{i=1}^{S}{C}_{i.}{\text{log}}\frac{{C}_{i.}}{N}+\sum_{j=1}^{R}{C}_{.j}{\text{log}}\frac{{C}_{.j}}{N}}$$

Suppose there are two networks denoted as ${Net}_{1}$ and ${Net}_{2}$ each consisting of sets of vertices (V) and edges (E). ${Net}_{1}$ consists of R communities denoted as $A=\{{A}_{1},{A}_{2},\dots .,{A}_{R}\}$, while ${Net}_{2}$ consists of S communities denoted as $B=\{{B}_{1},{B}_{2},\dots .,{B}_{S}\}$. ${C}_{ij}$ denotes the number of nodes that clusters (communities) ${A}_{i}$ and ${B}_{j}$ share. If $A=B$, then $NMI (A, B)=1$; if A and B are completely different, then $NMI (A,B)=0$.

In addition to the NMI, some other measures can be used to evaluate the accuracy of CD and clustering algorithms. Table 1 lists well-known measures in this domain. We listed these measures here to highlight the differences between the NMI and other measures in practice.

**Table 1:** Well-known metrics for evaluating community detection algorithms.

Metric	Formula	Symbol
Normalized Mutual Information (NMI)	$\frac{-2\sum_{i=1}^{S}\sum_{j=1}^{R}{C}_{ij}{\text{log}}\frac{{C}_{ij}N}{{C}_{i.}{C}_{.j}}}{\sum_{i=1}^{S}{C}_{i.}{\text{log}}\frac{{C}_{i.}}{N}+\sum_{j=1}^{R}{C}_{.j}{\text{log}}\frac{{C}_{.j}}{N}}$	*NMI*
Adjusted Rand Index	$\frac{\sum_{ij}\left(\genfrac{}{}{0pt}{}{{C}_{ij}}{2}\right)-\sum_{i}\left(\genfrac{}{}{0pt}{}{{C}_{i.}}{2}\right)\sum_{j}\left(\genfrac{}{}{0pt}{}{{C}_{.j}}{2}\right)/\left(\genfrac{}{}{0pt}{}{N}{2}\right)}{\frac{1}{2}\left[\sum_{i}\left(\genfrac{}{}{0pt}{}{{C}_{i.}}{2}\right)+\sum_{j}\left(\genfrac{}{}{0pt}{}{{C}_{.j}}{2}\right)\right]-\sum_{i}\left(\genfrac{}{}{0pt}{}{{C}_{i.}}{2}\right)\sum_{j}\left(\genfrac{}{}{0pt}{}{{C}_{.j}}{2}\right)/\left(\genfrac{}{}{0pt}{}{N}{2}\right)}$	*ARI*
Pairwise F measure	$\frac{2\times precision\times recall}{precision+recall}$	*PWF*
Fowlkes Mallows	$\frac{\sum_{ij}\left(\genfrac{}{}{0pt}{}{{C}_{ij}}{2}\right)}{\sqrt{\sum_{i}\left(\genfrac{}{}{0pt}{}{{C}_{i.}}{2}\right)\sum_{j}\left(\genfrac{}{}{0pt}{}{{C}_{.j}}{2}\right)}}$	*FM*
Hubert statistic	$\frac{\left(\genfrac{}{}{0pt}{}{N}{2}\right)\sum_{ij}\left(\genfrac{}{}{0pt}{}{{C}_{ij}}{2}\right)-\sum_{i}\left(\genfrac{}{}{0pt}{}{{C}_{i.}}{2}\right)\sum_{j}\left(\genfrac{}{}{0pt}{}{{C}_{.j}}{2}\right)}{\sqrt{\sum_{i}\left(\genfrac{}{}{0pt}{}{{C}_{i.}}{2}\right)\sum_{j}\left(\genfrac{}{}{0pt}{}{{C}_{.j}}{2}\right)\left[\left(\genfrac{}{}{0pt}{}{N}{2}\right)-\sum_{i}\left(\genfrac{}{}{0pt}{}{{C}_{i.}}{2}\right)\right][\left(\genfrac{}{}{0pt}{}{N}{2}\right)-\sum_{j}\left(\genfrac{}{}{0pt}{}{{C}_{.j}}{2}\right)]}}$	${\text{Hubert}}$
Jaccard	$\frac{\sum_{ij}\left(\genfrac{}{}{0pt}{}{{C}_{ij}}{2}\right)}{\sum_{i}\left(\genfrac{}{}{0pt}{}{{C}_{i.}}{2}\right)+\sum_{j}\left(\genfrac{}{}{0pt}{}{{C}_{.j}}{2}\right)-\sum_{ij}\left(\genfrac{}{}{0pt}{}{{C}_{ij}}{2}\right)}$	Jaccard

Essentially, to compute the measures listed in Table 1, a contingency table (CT) is employed. This table is created based on the joint members between communities detected by a certain algorithm and the ground truth. The contingency table used for computing the NMI is presented in Table 2.

**Table 2:** Contingency table.

S	R				
	${r}_{1}$	${r}_{2}$	…	${r}_{r}$	Sum
${s}_{1}$	${c}_{11}$	${c}_{12}$	…	${c}_{1r}$	${c}_{1.}$
${s}_{2}$	${c}_{21}$	${c}_{22}$	…	${c}_{2r}$	${c}_{2.}$
…	…	…		…	…
${s}_{s}$	${c}_{s1}$	${c}_{s2}$	…	${c}_{sr}$	${c}_{s.}$
Sum	${c}_{.1}$	${c}_{.2}$	…	${c}_{.r}$	

Table 3 presents important notations.

**Table 3:** Key notations.

Notion	Description
${{\varvec{C}}}_{{\varvec{i}}.}$	Refers to summation of cells in row i of Contingency table
${{\varvec{C}}}_{.{\varvec{j}}}$	Refers to summation of cells in column j of Contingency table
${{\varvec{C}}}_{{\varvec{i}}{\varvec{j}}}$	Refers to joint members in communities i and j
***R***	Refers to No. community which detected by certain algorithm
***S***	Refers to No. community in gold standard (ground truth)
${\varvec{\omega}}$	No. Ground truth with full same common members
${\boldsymbol{\varnothing }}^{{\varvec{S}}}$	Community in S

## Problem statement

In this section, we present the main drawback of the NMI. We illustrate this problem through an example. Example 1. Suppose we have 40 members and eight gold standard communities (ground truth) as follows:

$${\mathrm{\varnothing }}_{1}^{S}=\{{a}_{1},...,{a}_{5}\}$$, $${\mathrm{\varnothing }}_{2}^{S}=\{{a}_{6},...,{a}_{10}\}$$, $${\mathrm{\varnothing }}_{3}^{S}=\{{a}_{11},...,{a}_{15}\}$$, $${\mathrm{\varnothing }}_{4}^{S}=\{{a}_{16},...,{a}_{20}\}$$, $${\mathrm{\varnothing }}_{5}^{S}=\{{a}_{21},...,{a}_{25}\}$$, $${\mathrm{\varnothing }}_{6}^{S}=\{{a}_{26},...,{a}_{30}\}$$, $${\mathrm{\varnothing }}_{7}^{S}=\{{a}_{31},...,{a}_{35}\}$$, $${\mathrm{\varnothing }}_{8}^{S}=\{{a}_{36},...,{a}_{40}\}$$

**Figure 1:**
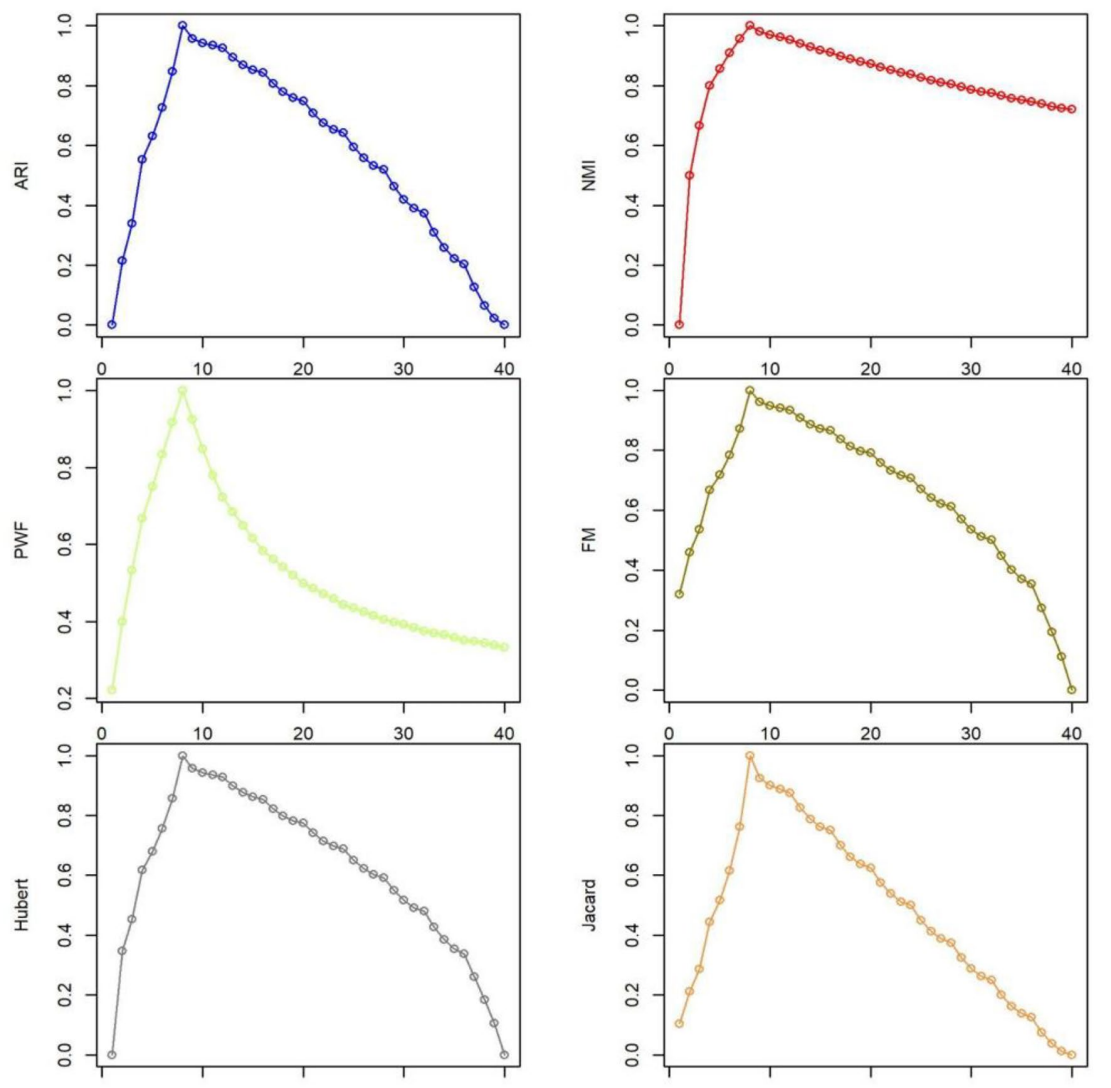
The response of different metrics based on varying numbers of communities.

We analyze all possible states based on the number of communities. Table 4 and Fig. 1 present the results of NMI values for different states compared to those of the ARI, PWF, Fowlkes Mallows, Hubert statistics, and Jaccard.

**Table 4:** The results of evaluation metrics based on all possible numbers of communities for a sample network with 40 nodes.

S	R	ARI	NMI	PWF	FM	Hubert	Jaccard	$\omega$
8	1	0	0	0.222	0.32	0	0.103	8
8	2	0.215	0.5	0.4	0.459	0.347	0.211	8
8	3	0.381	0.665	0.542	0.56	0.485	0.314	8
8	4	0.552	0.8	0.667	0.667	0.617	0.444	8
8	5	0.631	0.857	0.75	0.718	0.679	0.516	8
8	6	0.727	0.909	0.833	0.784	0.756	0.615	8
8	7	0.847	0.957	0.917	0.873	0.857	0.762	8
8	8	1	1	1	1	1	1	8
8	9	0.957	0.98	0.925	0.962	0.958	0.925	7
8	10	0.942	0.969	0.848	0.949	0.943	0.9	7
8	11	0.934	0.962	0.779	0.942	0.936	0.888	7
8	12	0.926	0.954	0.722	0.935	0.929	0.875	7
8	13	0.894	0.94	0.684	0.908	0.899	0.825	6
8	14	0.869	0.929	0.649	0.887	0.877	0.788	6
8	15	0.852	0.919	0.616	0.873	0.862	0.762	6
8	16	0.843	0.912	0.583	0.866	0.854	0.75	6
8	17	0.807	0.899	0.562	0.837	0.823	0.7	5
8	18	0.779	0.889	0.542	0.814	0.799	0.662	5
8	19	0.759	0.88	0.521	0.798	0.782	0.638	5
8	20	0.749	0.873	0.5	0.791	0.774	0.625	5
8	21	0.708	0.862	0.487	0.758	0.741	0.575	4
8	22	0.676	0.852	0.473	0.733	0.715	0.538	4
8	23	0.654	0.844	0.46	0.716	0.697	0.512	4
8	24	0.642	0.838	0.444	0.707	0.688	0.5	4
8	25	0.595	0.827	0.436	0.671	0.651	0.45	3
8	26	0.558	0.818	0.426	0.642	0.622	0.412	3
8	27	0.532	0.811	0.416	0.622	0.602	0.388	3
8	28	0.519	0.805	0.405	0.612	0.592	0.375	3
8	29	0.464	0.796	0.398	0.57	0.549	0.325	2
8	30	0.42	0.787	0.392	0.536	0.516	0.288	2
8	31	0.39	0.78	0.384	0.512	0.492	0.262	2
8	32	0.374	0.775	0.375	0.5	0.48	0.25	2
8	33	0.31	0.766	0.37	0.447	0.428	0.2	1
8	34	0.258	0.758	0.365	0.403	0.385	0.162	1
8	35	0.222	0.752	0.359	0.371	0.354	0.138	1
8	36	0.204	0.747	0.352	0.354	0.337	0.125	1
8	37	0.127	0.739	0.348	0.274	0.26	0.075	0
8	38	0.065	0.731	0.344	0.194	0.184	0.038	0
8	39	0.022	0.725	0.339	0.112	0.106	0.012	0
8	40	0	0.721	0.333	0	0	0	0

The number of communities in the ground truth is 8, and it should remain constant across all the experiments. The second column shows the number of communities detected by a specific algorithm, while columns 3–8 display the values for each measure. The last column represents the number of ground truth communities that share common members with the detected communities. For example, if a certain algorithm detects two communities as follows:

$${\varnothing }_{1}^{R}=\{{a}_{1},...,{a}_{5}\}, {\varnothing }_{1}^{R}=\{{a}_{6},...,{a}_{40}\}$$

The ${\varvec{\omega}}$ value is 8 since all members of the 8 ground truth communities share common members with the detected communities.

In this study, we assume the best-case scenario in which the detected communities of a certain algorithm lead to the highest NMI value. Thus, we propose:

***Axiom 1*** *: The highest NMI value is obtained if and only if the detected communities have the highest possible number of common members with the ground truth and if the highest value of* ${\varvec{\omega}}$ *is maintained.*

Now, as shown in Table 4 and Fig. 1, everything appears to be fine when the number of *R* is less than *S*. However, the situation changes when the number of *R* increases and surpasses S. At first glance, it may not seem that there is a strong argument to support the claim that the value of metrics declines as the number of members decreases. However, upon closer examination, it becomes evident that the decrease in the NMI value is less steep compared to the other metrics. This raises the question of what is amiss when comparing all algorithms to a specific metric such as the NMI.

The problem arises when N is equal to the number of communities, indicating that each community includes only one member, which is the worst-case scenario. Surprisingly, even in this unfavorable situation, the NMI value still indicated a high level of efficiency. On the other hand, when *R* equals one or two, the NMI value returned is very low. For instance, Table 4 demonstrates that when R is 2, the NMI is 0.5, whereas in the worst-case scenario with *R* being 40, the NMI is 0.72. This finding implies that if Alg1 and Alg2 return 2 and 40 communities respectively, the NMI suggests that the accuracies of Alg1 and Alg2 are 0.5 and 0.72 respectively. Similarly, when *R* is 4, the NMI is 0.8, whereas when *R* is 27 the NMI is also 0.8. In both examples, the NMI suggests that a certain algorithm that detects more communities is better. However, in reality, a certain algorithm that detects 4 communities may be better than another algorithm that detects 27 communities with respect to the number of ground truth communities.

## Proof of biased behavior of NMI

As discussed in the previous section, when the number of communities (*R*) increases and exceeds the number of communities in the ground truth (*S*), the NMI exhibits a biased behavior. Therefore, in this section, we aim to analyze the effect of quantity of communities on this measure. Firstly, we decompose the NMI formula, allowing us to examine how NMI values change from the minimum to the maximum. Subsequently, we present an explanation as to why NMI changes are more pronounced when $R<S$ rather than $R>S$. Before we begin our discussion, it should be noted that for all Lammas and the relevant proof, Axiom 1 should be maintained.

### Decomposition

For simplicity, we have Formula 4 (NMI) here.

$$NMI\left(A,B\right)=\frac{-2\sum_{i=1}^{S}\sum_{j=1}^{R}{C}_{ij}{\text{log}}\frac{{C}_{ij}N}{{C}_{i.}{C}_{.j}}}{\sum_{i=1}^{S}{C}_{i.}{\text{log}}\frac{{C}_{i.}}{N}+\sum_{j=1}^{R}{C}_{.j}{\text{log}}\frac{{C}_{.j}}{N}}$$

We decompose the above equation to examine the behavior and performance of each component. The NMI consists of three main components: the common members (${C}_{ij}$), the sum of common members for each community detected by a particular algorithm (${C}_{.j}$), and the sum of common members for each community in the ground truth (${C}_{i.}$). Here, *S* represents the number of communities in the ground truth, and *R* represents the number of communities detected by a specific algorithm. We denote the set of common members as *Z*. *S* represents the set of the sum of common members in each ground truth community, while *R* represents the set of common members in each community detected by a specific algorithm.

$$Z=\{{C}_{11},{C}_{12},\dots ,{C}_{21},{C}_{22},\dots {C}_{RS}\}$$

$S=\{{s}_{1},{s}_{2},\dots ,{s}_{s}\}$ is a set representing the number of members in each community of the ground truth, where ${s}_{i}$ denotes the number of members in community *i*. It is important to note that there exists an inverse relationship between each pair of elements in *S*.

$R=\{{r}_{1},{r}_{2},\dots ,{r}_{r}\}$ is a set representing the number of members in each community detected by a certain algorithm, where ${r}_{j}$ represents the number of members in community *j*. Again, there exists an inverse relationship between each pair of elements in *R*.

Suppose that:

5 $$K=\sum_{i=1}^{S}\sum_{j=1}^{R}{C}_{ij}{\text{log}}\frac{{C}_{ij}N}{{C}_{i.}{C}_{.j}}, L=\sum_{i=1}^{S}{C}_{i.}{\text{log}}\frac{{C}_{i.}}{N}, M=\sum_{j=1}^{R}{C}_{.j}{\text{log}}\frac{{C}_{.j}}{N}$$

6 $$\therefore NMI=\frac{-2K}{L+M}$$

The *L* and *M* values are always negative, ∵ $0<{C}_{i.}$ $<N \therefore$ ${\text{log}}\frac{{C}_{i.}}{N}$ $<0$ ⇒ ${C}_{i.}{\text{log}}\frac{{C}_{i.}}{N}$ $<0$

In addition, *K* is positive, therefore, the − 2 multiplied *K* is also negative and the NMI value is positive.

and:

7 $${s}_{i}=\sum_{k=1}^{S}{C}_{ik}={C}_{i}.\mathrm\,{And}\,{r}_{j}=\sum_{k=1}^{R}{C}_{kj}={C}_{.j}$$

#### Lemma 1

$$N={\sum }_{j=1}^{R}{r}_{j}={\sum }_{i=1}^{S}{s}_{i}$$

#### Proof

In simple terms, a certain algorithm detects communities with N members, where these N members are distributed among R communities. Similarly, this term holds true for the ground truth, which consists of S communities. Below, we provide a formal proof:

$$\begin{aligned} \forall i \exists {s}_{i} :{s}_{i}&={\sum }_{K=1}^{S}{C}_{ik},\& \,\forall \,S \,consist\, of\, |S| \,communities : {S}_{i}\, consist\, of\, elements\, in\, community\, i\, \therefore N=\sum {S}_{i}\,\\ \forall j \exists {r}_{j} : {r}_{j}&={\sum }_{k=1}^{R}{C}_{kj},\&\, \forall\, R\, consist\, of\, |R|\, communities : {R}_{j} \,consist\, of\, elements\, in\, community\, i\, \therefore N=\sum {R}_{j} \end{aligned}$$

### From minimum to maximum

#### Lemma 2

* The minimum value of NMI is obtained if* $R=1 and S>1 OR S=1 and R>1$

#### Proof

If $R=1$, then a certain algorithm considers all members in one community, on the other hand $S>1$ demonstrates that there is more than one community in ground truth. The conclusion is that ${C}_{ij}= {C}_{i.}$ and $N= {C}_{.j}$ therefore $K=0$. In a similar vein when $S=1$ and $R>1$ NMI is zero. Formally this can be shown as:

In both cases K is zero, ∵ ${C}_{ij}N{=C}_{i.}{C}_{.j}$ $\Rightarrow {\text{log}}1=0$, ∴ $NMI=0$.

#### ${\varvec{R}}={\varvec{S}}$

##### Lemma 3

*The maximum value of NMI is 1, if* $R=S \& \forall i, j, i=j \Rightarrow {C}_{ij}\ne 0 \& s.t i\ne j \Rightarrow {C}_{ij}=0$ *and the* *CT* *is a square and diagonal matrix*.

##### Proof

When $R=S$ only diagonal elements of CT is non zero, to have a maximum NMI value of 1, the members of each community in the ground truth first should be the same in terms of quantity and second in terms of member similarity with the communities detected by a certain algorithm. It should be noted that this does not imply that the number of members in all communities should be the same. Therefore, when R equals S, a square matrix is formed, ensuring that there are similar members in each pair. This results in members not being distributed across two communities, thus resulting in a diagonal matrix for the contingency table. Having only one non-zero cell leads to:

$$\forall i, j {C}_{ij}={C}_{i.}={C}_{.j}$$

$$\frac{{C}_{ij}N}{{C}_{i.}{C}_{j.}}=\frac{{C}_{i.}N}{{C}_{i.}{C}_{i.}}=\frac{N}{{C}_{i.}}={(\frac{{C}_{i.}}{N})}^{-1}$$

$$NMI=\frac{-2\sum_{i=1}^{S}\sum_{j=1}^{R}{C}_{i.}{\text{log}}\left(\frac{N}{{C}_{i.}}\right)}{\sum_{i=1}^{S}{C}_{i.}{\text{log}}\left(\frac{{C}_{i.}}{N}\right)+\sum_{i=1}^{S}{C}_{i.}{\text{log}}\left(\frac{{C}_{i.}}{N}\right)}$$

Since the CT is diagonal, only one cell in each row or column is considered to be non-zero ∴

8 $$\sum_{i=1}^{S}\sum_{j=1}^{R}{C}_{i.}{\text{log}}\left(\frac{N}{{C}_{i.}}\right)= \sum_{i=1}^{S}{C}_{i.}{\text{log}}\left(\frac{N}{{C}_{i.}}\right)$$

In addition:

9 $$\sum_{i=1}^{S}{C}_{i.}{\text{log}}\frac{{C}_{i.}}{N}= \sum_{j=1}^{R}{C}_{.j}{\text{log}}\frac{{C}_{.j}}{N}$$

∴

$$\therefore (8)(9)\Rightarrow \frac{-2 \sum_{i=1}^{S}{C}_{i.}{\text{log}}\left(\frac{N}{{C}_{i.}}\right) }{2 \sum_{i=1}^{S}{C}_{i.}{\text{log}}\frac{{C}_{i.}}{N} }=\frac{-2 \sum_{i=1}^{S}{C}_{i.}{\text{log}}\frac{N}{{C}_{i.}}}{-2 \sum_{i=1}^{S}{C}_{i.}{\text{log}}\frac{N}{{C}_{i.}} }=1$$

Furthermore, this can be demonstrated through *contradiction* by considering the case when *L* and *M* are not equal, therefore $R\ne S$. If $R>S$, it implies that some members are distributed across different communities. This distribution decreases the number of common members, consequently increasing the absolute value of the denominator in the fraction. Consequently, the NMI decreases, as shown in Eqs. (10) and (11):

10 $$\mathrm{Where}\,a=b+c\mathrm\,{ then }|{\text{log}}\frac{a}{N}|<|{\text{log}}\frac{b}{N}|+|{\text{log}}\frac{c}{N}|$$

Now, if $S>R$, the absolute value of the numerator decreases. This occurs because the logarithmic reduction has a smaller slope compared to the effect of the coefficient.

11 $$a|{\text{log}}\frac{a}{N}|<b|{\text{log}}\frac{b}{N}|+c|{\text{log}}\frac{c}{N}|\,\mathrm{Where}\,a>N$$

In the given example, the roles of *K* and *M* are crucial in the NMI formula, where *L* is constant. Let us consider the two algorithms, Alg 1 and Alg 2, along with their respective communities.

##### Example 2

Suppose the ground truth consists of 4 communities: $\{{a}_{1},{a}_{2}\},\{{a}_{3},{a}_{4}\},\{{a}_{5},{a}_{6}\},\{{a}_{7},{a}_{8}\}$

Algorithm Alg 1 consists of two communities: $\{{a}_{1},{a}_{2},{a}_{3},{a}_{4}\},\{{a}_{5},{a}_{6},{a}_{7}{,a}_{8}\}$. In this case, the values for *K, L, M*, and NMI for Alg 1 are as follows:

$$K=2.76, L=-\mathrm{11,09}, M=-\mathrm{5,54}\,and\, NMI=0.66$$

Now, consider Algorithm Alg 2, which consists of six communities:$\{{a}_{1},{a}_{2}\},\{{a}_{3},{a}_{4}\},\{{a}_{5}\},\{{a}_{6}\},\{{a}_{7}\},{\{a}_{8}\}$. In this case, the values for *K, L, M*, and NMI for Alg 2 are as follows:

$$K=\mathrm{11,09}, L=-\mathrm{11,09}, M=-\mathrm{13,86}\,and\, NMI=0.88$$

These values demonstrate the roles of K and M in the NMI formula, and how they contribute to the calculation of the NMI value for Alg 1 and Alg 2, respectively.

#### ${\varvec{R}}\ne {\varvec{S}}$

Now, let us discuss the scenarios where the number of communities is greater or less than the number of communities in the ground truth.

- $${\varvec{R}}>{\varvec{S}}$$

It is clear that $|{\varvec{Z}}|={\varvec{R}}\times {\varvec{S}}$

For this state:

##### Lemma 4

* If* $R>S$, *then the maximum value of NMI is obtained if and only if the cardinality of the set of common members (Z*) *is equal to* ${\varvec{R}}\times ({\varvec{R}}-1)$, *and if the number of pairwise common members* (${C}_{RS}$) *that are not equal to zero is equal to or greater than the cardinality of* ${\varvec{R}}$.

First it is important to note that values smaller than |R|, indicating non-zero values in the cells of the contingency table, are not possible. This is because having a community without any common members violates the *pigeonhole principle* in mathematics. According to this principle, if *n* items are placed into *m* containers, where $n>m$, then at least one container must contain more than one item.

In this scenario, at least one detected community should have at least one common member, resulting in at least |*R*| being the non-zero member in *Z*. This can be illustrated with an example.

##### Example 3

Let us suppose that the ground truth has 2 communities, and a certain algorithm detects 3 communities as follows:

$${\varnothing }_{1}^{S}=\{{a}_{1},{a}_{2}, {a}_{3}, {a}_{4}\},{\varnothing }_{2}^{S}=\{{a}_{5},{a}_{6, }{a}_{7}, {a}_{8}\}$$

$${\varnothing }_{1}^{R}=\{{a}_{1},{a}_{2},{a}_{3},{a}_{4}\}, {\varnothing }_{2}^{R}=\{{a}_{5},{a}_{6}\}, {\varnothing }_{3}^{R}=\{{a}_{7},{a}_{8}\}$$

Table 5 displays the number of common members (*Z*). The number of nonzero elements in *Z* is 3, which is equal to or greater than |*R*|.

**Table 5:** Contingency table of the sample community detection algorithm.

	${r}_{1}$	${r}_{2}$	${r}_{3}$
${s}_{1}$	4	0	0
${s}_{2}$	0	2	2

##### Proof (lemma 4)

The greater the difference between R and S, the greater the number of communities detected. This results in fewer common members between the communities in R and S, subsequently leading to a decrease in NMI. Additionally, if the number of non-zero members in Z is greater than R, it indicates that the number of common members between S and R is less, further contributing to the reduction in NMI.

- $${\varvec{R}}<{\varvec{S}}$$

For this state:

##### Lemma 5

* If* ${\varvec{R}}<{\varvec{S}}$, *then the maximum value of NMI is obtained if and only if the cardinality of the set of common members (Z) is equal to* ${\varvec{S}}\times ({\varvec{S}}-1)$, *and if the number of pairwise common members* (${C}_{RS}$) *that are not equal to zero is equal to the cardinality of S*.

Like Lemma 4, Lemma 5 can be proven.

### Slope of change in the NMI

#### Lemma 6

* When the difference between* *R* *and* *S* *increases, in cases where* $R < S$, *the slope of changes of the NMI values are greater than when* $R>S$.

#### Proof

By applying Lemma 4 and Lemma 5, we can demonstrate that if there is a difference between *R* and *S*, the optimal NMI value is obtained when the difference between *R* and *S* is 1. In other words, as the difference between *R* and *S* increases, the NMI decreases.

Equation (6) is equal to function 12 where we consider $NMI$ as $f(x,y, z)$, $K=g(x,y,z), M=h(z)$:

s.t. $$x={C}_{ij} , y={C}_{i.} , z={C}_{.j}$$

12 $$f\left(x,y,z\right)=NMI=\frac{-2g(x,y,z)}{L+h(z)}$$

$$L=\sum_{i=1}^{S}{C}_{i.}{\text{log}}(\frac{{C}_{i.}}{N})=\sum_{i=1}^{S}{C}_{i.}{\text{log}}\left({C}_{i.}\right)-\sum_{i=1}^{S}{C}_{i.}{\text{log}}N= \sum_{i=1}^{S}{C}_{i.}{\text{log}}\left({C}_{i.}\right)-N{\text{log}}N$$

$$=\left({C}_{1.}{\text{log}}\left({C}_{1.}\right)+{C}_{2.}{\text{log}}\left({C}_{2.}\right)+\dots +{C}_{S.}{\text{log}}\left({C}_{S.}\right)\right)-N{\text{log}}N$$

where $$O=({C}_{1.}{\text{log}}\left({C}_{1.}\right)+{C}_{2.}{\text{log}}\left({C}_{2.}\right)+\dots +{C}_{S.}{\text{log}}({C}_{S.}))$$

13 $$L=O-N{\text{log}}N$$

$$h\left(z\right)=\sum_{j=1}^{R}{C}_{.j}{\text{log}}(\frac{{C}_{.j}}{N})=\sum_{j=1}^{R}{C}_{.j}{\text{log}}\left({C}_{.j}\right)-\sum_{j=1}^{R}{C}_{.j}{\text{log}}N=\sum_{j=1}^{R}{C}_{.j}{\text{log}}\left({C}_{.j}\right)-N{\text{log}}N$$

$$=\left({C}_{.1}{\text{log}}\left({C}_{.1}\right)+{C}_{.2}{\text{log}}\left({C}_{.2}\right)+\dots +{C}_{.R}{\text{log}}\left({C}_{.R}\right)\right)-N{\text{log}}N$$

14 $$\mathrm{where }\,P=({C}_{.1}{\text{log}}\left({C}_{.1}\right)+{C}_{.2}{\text{log}}\left({C}_{.2}\right)+...+{C}_{.R}{\text{log}}({C}_{.R}))$$

15 $$h\left(z\right) =P-N{\text{log}}N$$

For all cases, $N{\text{log}}N\mathrm{\,is\, a\, constant},\mathrm{\,therefore\,}h(z)\mathrm{\,is\, completely\, dependent\, on\, }P,\,\mathrm{s}.\mathrm{t}\,P<N{\text{log}}N$

When $S>R$, increasing *R* results in a decrease in *P*, causing $|h(z)|$ to increase. This might suggest that *NMI* decreases. However, $g(x,y,z)$ increases, leading to an increase in *NMI*. Let us examine $g(x,y,z)$ to further understand this.

$$g\left(x,y,z\right)=\sum_{i=1}^{S}\sum_{j=1}^{R}{C}_{ij}{\text{log}}(\frac{{C}_{ij}N}{{C}_{i.}{C}_{.j}})=\sum_{i=1}^{S}\sum_{j=1}^{R}{C}_{ij}{\text{log}}\left({C}_{ij}N\right)-\sum_{i=1}^{S}\sum_{j=1}^{R}{C}_{ij}{\text{log}}\left({C}_{i.}{C}_{.j}\right)$$

$$=\sum_{i=1}^{S}\sum_{j=1}^{R}{C}_{ij}{\text{log}}\left({C}_{ij}\right)+\sum_{i=1}^{S}\sum_{j=1}^{R}{C}_{ij}{\text{log}}\left(N\right)-\sum_{i=1}^{S}\sum_{j=1}^{R}{C}_{ij}{\text{log}}\left({C}_{i.}\right)-\sum_{i=1}^{S}\sum_{j=1}^{R}{C}_{ij}{\text{log}}\left({C}_{.j}\right)$$

16 $$=\sum_{i=1}^{S}\sum_{j=1}^{R}{C}_{ij}{\text{log}}\left({C}_{ij}\right)+N{\text{log}}N-\sum_{i=1}^{S}\sum_{j=1}^{R}{C}_{ij}{\text{log}}\left({C}_{i.}\right)-\sum_{i=1}^{S}\sum_{j=1}^{R}{C}_{ij}{\text{log}}\left({C}_{.j}\right)$$

17 $$\sum_{i=1}^{S}\sum_{j=1}^{R}{C}_{ij}{\text{log}}\left({C}_{i.}\right)=\sum_{i=1}^{S}{C}_{i.}{\text{log}}({C}_{i.})=O$$

18 $$\sum_{i=1}^{S}\sum_{j=1}^{R}{C}_{ij}{\text{log}}\left({C}_{.j}\right)=\sum_{j=1}^{R}{C}_{.j}{\text{log}}({C}_{.j})=P$$

19 $$\left(16\right), \left(17\right), and\, \left(18\right)\Rightarrow {\text{g}}\left({\text{x}},{\text{y}},{\text{z}}\right)=\sum_{i=1}^{S}\sum_{j=1}^{R}{C}_{ij}{\text{log}}{C}_{ij}+N{\text{log}}N-P-O$$

$$\left(12\right), \left(13\right),\left(15\right){\text{\,and}}\,(19)\Rightarrow {\text{NMI}}=\frac{-2(\sum_{i=1}^{S}\sum_{j=1}^{R}{C}_{ij}{\text{log}}{C}_{ij}+N{\text{log}}N-P-O)}{O+P-2 (N{\text{log}}N)}$$

$$=\frac{-2\sum_{i=1}^{S}\sum_{j=1}^{R}{C}_{ij}{\text{log}}{C}_{ij}-2(N{\text{log}}N-P-O)}{O+P-2 (N{\text{log}}N)}=\frac{-2\sum_{i=1}^{S}\sum_{j=1}^{R}{C}_{ij}{\text{log}}{C}_{ij}+2(O+P-N{\text{log}}N)}{O+P-2(N{\text{log}}N)}$$

suppose $$\sum_{i=1}^{S}\sum_{j=1}^{R}{C}_{ij}{\text{log}}{C}_{ij}=T$$ then

20 $$=\frac{2\left(O+P\right)-2 N{\text{log}}N -2T}{O+P-2 N{\text{log}}N}$$

*O* and $N{\text{log}}N$ are constant for all scenarios. Let us suppose *O* = ${C}_{1}$ and $2 N{\text{log}}N$= ${C}_{2}$:

21 $$(20)\Rightarrow NMI=\frac{2({C}_{1} +P)-{C}_{2}-2T}{{C}_{1} +P-{C}_{2}}$$

Now, let us consider Eq. (21) for two possible scenarios:

1. $R<S$ (the number of detected communities is lower than that of the ground truth):

In this scenario, let us suppose that Algorithm 1 returns ${N}_{1}$ communities in one run and ${N}_{2}$ communities in another run, where ${N}_{1} <{N}_{2}.$. According to *Axiom 1*, as the number of communities increases, the possibility of member distribution also increases, which leads to a reduction in the *P* value according to (22) (14) and (13). A decrease in *P* results in a larger numerator in Eq. (21). This is because it increases the difference with ${C}_{2}$, where ${C}_{2}$, is a constant value for all the scenarios.

∵

$$Na=\frac{Na}{2}+\frac{Na}{2}$$

$$N{\text{log}}a >\frac{N}{2}{\text{log}}\frac{a}{2}+\frac{N}{2}{\text{log}}\frac{a}{2}$$

$$\because N{\text{log}}a>\frac{N}{2}{\text{log}}a-\frac{N}{2}{\text{log}}2+\frac{N}{2}{\text{log}}a-\frac{N}{2}{\text{log}}2$$

22 $$N{\text{log}}a>N{\text{log}}a-N{\text{log}}2$$

For Alg 1 $${R}_{1}=\{{r}_{{1}_{.1}},{r}_{{1}_{.2}},...,{r}_{{1}_{.{N}_{1}}}\}$$

For Alg 2, $${R}_{2}=\{{r}_{{2}_{.1}},{r}_{{2}_{.2}},...,{r}_{{2}_{.{N}_{2}}}\}$$

$${N}_{2}>{N}_{1}: \frac{N}{{N}_{2}}<\frac{N}{{N}_{1}}$$ ∴$$\forall i {r}_{{2}_{.i}}\le {r}_{{1}_{.i}}\&\nexists j {r}_{{2}_{.j}}>{r}_{{1}_{.j}} \& \exists k {r}_{{2}_{.k}}<{r}_{{1}_{.k}}$$

∴ According to (14) and (22) $P1>P2$

To further illustrate this, let us consider the following  example:

#### Example 4

Suppose the ground truth consists of 4 communities, Algorithm 1 returns 2 communities, Algorithm 2 returns 3 communities (in both cases, $R<S$), and *N* = 8.

$${\varnothing }_{1}^{S}=\{{a}_{1},{a}_{2}\},{\varnothing }_{2}^{S}=\{{a}_{3},{a}_{4}\},{\varnothing }_{3}^{S}=\{{a}_{5},{a}_{6}\},{\varnothing }_{4}^{S}=\{{a}_{7},{a}_{8}\}$$

$${\varnothing }_{1}^{Alg1}=\{{a}_{1},{a}_{2},{a}_{3},{a}_{4}\}, {\varnothing }_{2}^{Alg1}=\{{a}_{5},{a}_{6},{a}_{7},{a}_{8}\}$$

$${\varnothing }_{1}^{Alg2}=\{{a}_{1},{a}_{2}\}, {\varnothing }_{2}^{Alg2}=\{{a}_{3},{a}_{4}\},{\varnothing }_{3}^{Alg2}=\{{a}_{5},{a}_{6},{a}_{7},{a}_{8}\}$$

Table 6 displays the contingency table of Alg1.

**Table 6:** Contingency table of Alg1.

S	R		
	${r}_{1}$	${r}_{2}$	${C}_{i.}$
${s}_{1}$	2	0	2
${s}_{2}$	2	0	2
${s}_{3}$	0	2	2
${s}_{4}$	0	2	2
${C}_{.j}$	4	4	

$${P }_{Alg1}=4log(4)+4log(4)\approx 11.09$$

$${O }_{Alg1}=2log(2)+2log(2)+2log(2)+2log(2)\approx 5.54$$

$${T}_{Alg1}=2log(2)+2log(2)+2log(2)+2log(2)\approx 5.54$$

$${NMI }_{Alg1}\approx \frac{33.26-2(5.54)-{C}_{2}}{16.63-{C}_{2}}\approx \frac{22.18-{C}_{2}}{16.63-{C}_{2}}\approx \frac{22.18-33.27}{16.63-33.27}\approx \frac{-11.09}{-16.64}\approx 0.66$$

Table 7 presents the contingency table of Alg 2.

**Table 7:** Contingency table of Alg 2.

S	R			
	${r}_{1}$	${r}_{2}$	${r}_{3}$	${C}_{i.}$
${s}_{1}$	2	0	0	2
${s}_{2}$	0	2	0	2
${s}_{3}$	0	0	2	2
${s}_{4}$	0	0	2	2
${C}_{.j}$	2	2	4	

$${P }_{Alg2}=2log(2)+2log(2)+4log(4)\approx 8.31$$

$${O }_{Alg2}=2log(2)+2log(2)+2log(2)+2log(2)\approx 5.54$$

$${T}_{Alg2}=2log(2)+2log(2)+2log(2)+2log(2)\approx 5.54$$

$${NMI }_{Alg2}\approx \frac{27.7-2(5.54)-{C}_{2}}{13.86-{C}_{2}}\approx \frac{16.61-33.27}{13.86-33.27}\approx \frac{16.66}{19.41}\approx 0.85$$

Therefore, the NMIs of different algorithms depend on *P* and *T*. Let us consider Alg1 and Alg2 with ${P}_{1}$, ${T}_{1}$, ${P}_{2}$, and ${T}_{2}$ respectively . Suppose Alg1 has a lower number of communities than Alg2. In this case, it can be proven that Eq. (23) holds.

23 $$\frac{2({C}_{1} +P)-{C}_{2} -2T}{{C}_{1} +P-{C}_{2} }<\frac{2({C}_{1} +P)-{C}_{2} -2T}{{C}_{1} +P-{C}_{2}}$$

1. R > S (the number of detected communities is greater than that of the ground truth)

To analyze the scenario where $R>S$, we start by incrementing R by one unit. This implies that, after the case where $R=S$, in the next state, a certain algorithm returns $R+1$ communities. As a reminder, S is constant. In this case, one of the community members is distributed across two communities to maintain Axiom 1. This can be expressed as:

$${C}_{SR-new}=[\frac{{C}_{SR-old}}{2}], {C}_{S,R+1}={C}_{SR-old}-[\frac{{C}_{SR-old}}{2}]$$

By further increasing *R*, this process is repeated until the new community consists of only one member. The distribution of shared members from one community to new communities results in a lower value of *P* compared to previous states according to (22) and (14). Additionally, $P=T$ in this case ($R>S$), the numerator remains constant for all scenarios.

The key issue here is that increasing R results in a non-linear (approximately logarithmic) decrease in P. The maximum value of P with respect to R is achieved if:

24 $$R=1 \Rightarrow {P}_{1}\approx N{\text{log}}N$$

25 $$R=2 \Rightarrow {P}_{2}\approx \frac{N}{2}{\text{log}}\frac{N}{2}+\frac{N}{2}{\text{log}}\frac{N}{2}$$

….

26 $$R=S \Rightarrow {P}_{R}\approx \sum_{j=1}^{R}\frac{N}{S}{\text{log}}\frac{N}{S}$$

…..

27 $$R=N\Rightarrow {P}_{N}\approx \sum_{j=1}^{R}\frac{N}{N}{\text{log}}\frac{N}{N}=0$$

First, according to (22)

$${P}_{1}>{P}_{2}>....>{P}_{n}$$

and

28 $${\forall i\ge 1, i<S \exists j\ge S\Rightarrow (P}_{i}-{P}_{i+1})>{(P}_{j}-{P}_{j+1})$$

According to Eq. (28), we can conclude that when $R<S$, the slope of the change curve is greater than when $R>S$. This results in a larger *P* value when $R<S$ compared to states where $R>S$. Additionally, it can be proven that the *T* value when $R<S$ is constant. Therefore, NMI when $R<S$ changes with respect to *P*, and a greater change in *P* when $R<S$ leads to a greater change in NMI.

**Figure 2:**
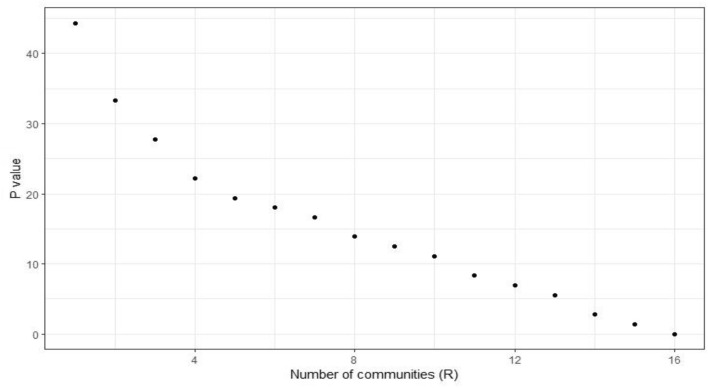
Nonlinear (approximately logarithmic) decreases in the *P* value.

Figure 2 illustrates a nonlinear (approximately logarithmic) decrease in the *P* value as the number of communities (R) increases.

#### Lemma 7

* When* $R<S$ *and Axiom 1 holds, T is a constant*.

#### Proof

$$\forall i,\exists j=k {C}_{ij}\ne 0\, \&\, \forall k\ne j {C}_{ik}=0\Rightarrow {C}_{ij}={C}_{i.}$$

$$\therefore T=O \Rightarrow T\, is\, constant$$

As the number of communities (*R*) becomes larger than the number of communities in the ground truth (*S*), the changes in P decrease. In this case, the value of *T* is equal to *P*, resulting in a constant numerator for the scenario when $R>S$. However, due to a small change in the denominator, the magnitude of change in NMI is smaller when $R>S$ than when $R<S$.

#### Lemma 8

*When* $R>S$ *and Axiom 1 holds, then T equals P.*

#### Proof

When $R=S$, the contingency table becomes a scalar matrix.

$$\forall i \exists j, s.t \,where\, i=j \,{then\, C}_{ij}\ne 0, where\, i\ne j {C}_{ij}=0$$

By increasing each unit in *R* when $R>S$ then:

$$\exists i \exists j \exists k {s.t C}_{i.}\cap {C}_{.j}\ne \varnothing ,{C}_{i.}\cap {C}_{.k}\ne \varnothing and \forall m s.t m\ne k,j{C}_{i.}\cap {C}_{.m}=\varnothing$$

$$\therefore {C}_{ij}={C}_{.i}\Rightarrow T=P$$

Regarding the values of (24) to (27), for the case when $R>S$, the *T* value is equal to *P*, resulting in a constant numerator for this scenario.

Overall, when we summarize the NMI formula in Eq. (21), the logarithm function remains the key factor. One of the main characteristics of the logarithm function is its low slope change with respect to its variable. This characteristic is reflected in the descending derivative of the logarithm function. This observation forms the main intuition behind Eq. (28), which explains the biased behavior of NMI. Furthermore, this phenomenon can be generalized to other entropy-based metrics that utilize the logarithm function in their formulas.

## Case study

**Figure 3:**
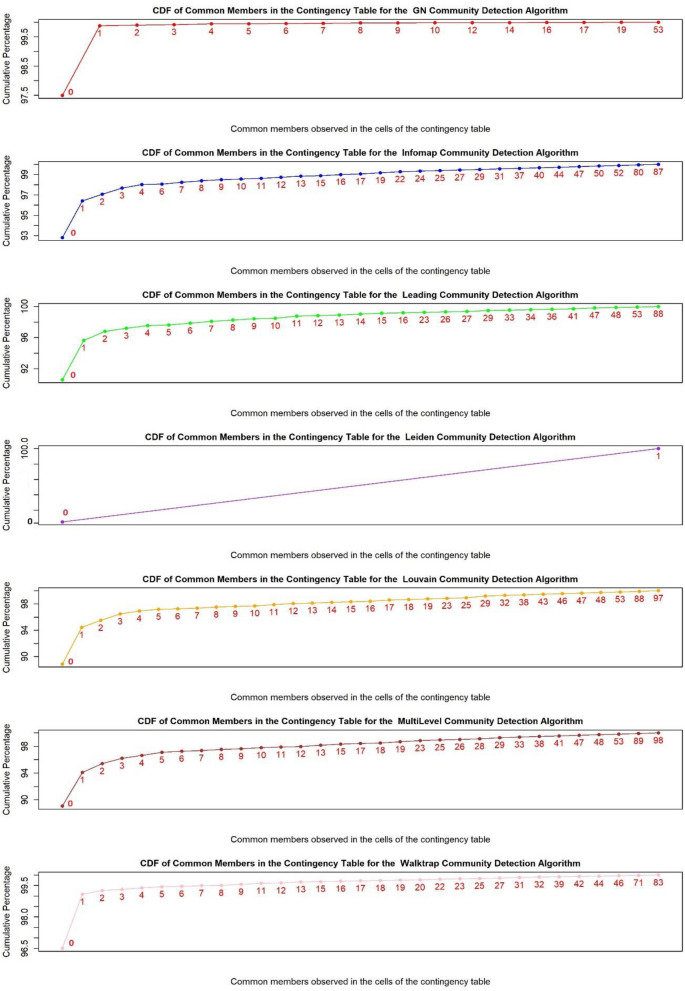
Cumulative Distribution Function (CDF) of common members based on CT.

In this section, we provide a case study to demonstrate why and how NMI returns biased results in practice. To do this, we utilized the email-Eu-core network dataset^50^, which was generated using email data from a large European research institution. This dataset is provided by Jure Leskovec from Stanford university^51^, and according to^50-p^^.^^12^ " we have anonymized information about all incoming and outgoing email of the research institution”. It consists of 1005 nodes (N = 1005) and 25,571 edges. To compare the NMI value and reveal its biased behavior in different scenarios according to R value, seven well-known community detection algorithms were deployed, including multi-level, Louvain, leading eigenvector, infomap, walktrap, edge betweenness (GN), and Leiden. These algorithms are implemented through specific functions developed to detect communities within the 'igraph' package of the R programming language. The functions corresponding to the aforementioned algorithms are as follows: multilevel.community, cluster_louvain, cluster_leading_eigen, cluster_infomap, cluster_walktrap, cluster_edge_betweenness, and cluster_leiden. These functions take as input a network comprising nodes and edges and return the node name along with its corresponding community number. Interestingly, a diverse range of R values obtained by the above-mentioned algorithms helps us clearly show the limitations of NMI. Additionally, a plot of the cumulative distribution function (CDF) of the percentage of common members according to CT is illustrated for each community detection algorithm (see Fig. 3). Each point in this plot is labeled with a number representing the number of common members. For instance, if a point is labeled 1 and the corresponding value on the y-axis is 97, it means that 97% of the cells in CT have 1 or fewer common members. The labels '0' and corresponding CDF values on the y-axis represent the percentages of cells in the CT with a zero value. This indicates a lack of common members between the communities detected by a certain algorithm and the communities in the ground truth. The x-axis illustrates the common members observed in the cells of the CT. For example, in Fig. 3's top plot, the CDF of common members in the contingency table for the GN algorithm reveals the occurrence of values 0, 1, 2, 3, 4, 5, 6, 7, 8, 9, 10, 12, 14, 16, 17, 19, and 53. These findings suggest that these values represent the number of common members between the communities detected by the GN algorithm and the ground truth. The CDF essentially shows the frequency of these values in the CT. It is evident that lower values are more commonly observed in the cells of the CT, with the exception of the Leiden algorithm. As we will discuss later, the Leiden algorithm returns a CT in which all cells are filled with the value '1'."

Table 8 shows the NMI values obtained by applying the above-mentioned algorithms to the email-Eu-core network. The abnormal NMI value, which alone can highlight the drawback of the NMI measure, is obtained by the Leiden algorithm, with a value of 0.65. Leiden returns 1005 communities, meaning that each community consists of only one node. In practice, this is a worst-case scenario, as considering each node as a separate community in relation to the ground truth (42 communities) is a noticeable fault.

**Table 8:** NMI values based on seven well-known community detection algorithms applied to the email-Eu-core network dataset.

Algorithm	R	S	P	O	NMI	ARI
Louvain	27	42	4840	3613	0.59	0.34
multi-level	27	42	4847	3613	0.58	0.32
Leading eigenvector	41	42	4718	3613	0.52	0.26
infomap	43	42	4500	3613	0.65	0.30
WalkTrap	145	42	4317	3613	0.58	0.20
Edge betweenness	731	42	1305	3613	0.60	0.06
Leiden	1005	42	0	3613	0.65	0

Furthermore, as shown in Fig. 3, 100% of the cells in CT for the Leiden algorithm have a value of 1, indicating that the communities detected by Leiden have only one common member with the ground truth communities. It is evident that the NMI value of 0.65 is a significantly biased value, whereas the ARI is zero for this algorithm, providing a more realistic measure of common members with the ground truth. The problem arises when an analyst deploys only the NMI without considering information about R, S, or common members, which is a common approach in scientific experiments in community detection studies. In such cases, the analyst may interpret Leiden or infomap as the best algorithm, while Leiden is actually the worst.

Now, let us consider the second-highest value of R in Table 8, which is the result of the edge betweenness (GN) algorithm. Its R value is 731, which is significantly greater than S, leading to the distribution of members in numerous communities. Consequently, it is less likely that the communities detected by GN align with the ground truth. However, the NMI for this algorithm is 0.6, which is a high value compared to that of the other algorithms in this case study and an inaccurate result. According to Fig. 3, approximately 97.5% of the CT cells are zero, more than 99% are 1 or 0, and only 0.5% of the cells are greater than 1.

Comparing Leiden and GN to Louvain highlights the incompetence of NMI. There are 27 communities returned by Louvain, suggesting a greater likelihood of having more common members with the ground truth. However, this is not guaranteed, as meeting Axiom 1 in a real-world scenario is not assured. Investigating common members based on the CDF plot of the number of cells depicted in Fig. 3 demonstrates that the Louvain algorithm leads to more similar communities with ground truth communities. Despite this fact, NMI returned higher values for GN and Leiden than for Louvain. It can be concluded that when R > S, NMI exhibits biased behavior. A comparison with other algorithms also highlights this problem.

Another interesting issue we find from this case study is how Eq. (28) behaves when R > S and R < S. As expressed in the explanation of Eq. (28), the changes in each pair of P when R < S are greater than when R > S. For instance, when R is 27, P is 4847, when R is 41, P is 4718, and the difference in P is 129. On the other hand, the difference in P when R is 43 and 145 is 183. Therefore, the changes in the case of R < S per unit are 129/(41–27) = 9.21, which is greater than when R > S, where the difference per unit is 183/(145–43) = 1.79. Therefore, it leads to a sharper change per unit in the NMI value when R < S, which is (0.58–0.52)/(41–27) × 100 = 0.43, compared to R > S, which is (0.65–0.58)/102 ×  100 = 0.07. This computation can be extended to other pairs of R values (e.g., 145, 731, and 1005) with respect to their P value. However, a meaningful comparison will be achieved if we have all possible R values, but in the real world, it is not an easy task to achieve this due to the limited number of community detection algorithms and the large number of nodes. Therefore, by considering a percentage and simulating it per unit, we provide an estimation of this problem.

### Ethical approval

The authors declare that there is no any human or live human cells involved in the study.

## Conclusion

In this study, we conducted an in-depth analysis of one of the most recognized measures employed in the evaluation of clustering and community detection algorithms. Utilizing a mathematical approach, we demonstrated the inherent bias of this measure (NMI), a bias that becomes particularly pronounced when the number of communities detected by a given algorithm surpasses the number of communities present in the ground truth. Our findings underscore the significant impact that the number of detected communities can have on each evaluation metric, an effect that is especially notable in logarithmic entropy-based metrics such as NMI. This observation is critical because it highlights the potential for skewed results and misinterpretations when using this metric in different applications.

The findings of this study can be generalized and applied in various contexts that utilize community detection evaluation metrics, ranging from friendship networks to critical applications such as identifying certain cancer diseases in protein–protein interaction (PPI) networks. Our study has highlighted and formally proven that the NMI is a biased metric for evaluating the results of community detection algorithms in any application. For instance, an algorithm that fails to detect the protein structure in PPI networks may be considered successful based on the NMI, potentially leading to the misdiagnosis of cancer. Therefore, our findings underscore the need for careful consideration of the characteristics and limitations of NMI, particularly in scenarios where the number of detected communities is high.Therefore, this study formally indicates that in the future, any field of study intending to base decisions on community detection algorithms should exercise caution in selecting the appropriate metric for evaluating these algorithms. This applies across various domains, such as marketing, where accurately targeting communities is crucial, or in information diffusion methods, where identifying dense communities is vital for activating more nodes.

However, the primary contribution of this study lies in revealing the root cause of this biased behavior, originating from the logarithmic function and its corresponding derivative. This insight holds significant value for future studies focused on designing new and equitable metrics within this domain. Understanding the mathematical behavior of this logarithmic metric can substantially aid in the creation of more precise evaluation metrics.

## Data Availability

The data and codes used and/or analyzed during the current study available from the corresponding author on request.
